# End-of-Life Care Provided to Patients and Families Who Die Shortly After Arriving at the Emergency Room: A Scoping Review

**DOI:** 10.7759/cureus.86705

**Published:** 2025-06-24

**Authors:** Ayako Fukushima, Yusuke Oyama, Megumi Horinouchi, Mayumi Koyanagi, Megumi Mukoyama, Megumi Kamogawa, Shun Yoshihara, Hideaki Sakuramoto

**Affiliations:** 1 Faculty of Health Sciences, Hokkaido University of Science, Sapporo, JPN; 2 Department of Nursing, Nagasaki University Graduate School of Biomedical Sciences, Nagasaki, JPN; 3 Department of Nursing, Hospital of the University of Occupational and Environmental Health, Kitakyushu, JPN; 4 Department of Nursing, Fukuoka Tokushukai Hospital, Kasuga, JPN; 5 Department of Nursing, Japanese Red Cross Fukuoka Hospital, Fukuoka, JPN; 6 Faculty of Nursing, Japanese Red Cross Kyushu International College of Nursing, Munakata, JPN; 7 Division of Faculty Development, Department of Nursing, Kindai University, Osakasayama, JPN

**Keywords:** emergency nursing, emergency room visits, palliative medicine, sudden death, terminal care

## Abstract

With the global aging population and rising mortality rates, more deaths are occurring in emergency rooms (ERs). Although ERs are primarily designed for acute treatment and resuscitation, they are also settings for end-of-life care. However, delivering high-quality end-of-life care in the ER is challenging owing to sudden deaths, limited resources, time constraints, and often unclear patient preferences. While end-of-life care aims to facilitate a “good death,” little is known about care practices for patients who die shortly after ER arrival. In this study, we aimed to clarify the content, barriers, and facilitators of end-of-life care in the ER. We systematically searched PubMed, Cumulative Index in Nursing and Allied Health Literature (CINAHL), and Ichushi-Web for studies published until November 22, 2024, using keywords related to ERs, death, and end-of-life care. The inclusion criteria included studies involving patients who died in the ER (without intensive care unit (ICU) admission or transfer) and end-of-life care provided in the ER setting, including perspectives from patients and families. Findings were integrated using a narrative synthesis approach, based on meaning-driven coding and categorization. Rather than formal thematic analysis, synthesis was guided by meaning-based classification and consensus among authors. A total of 3,614 studies were screened. Of 125 identified studies, 46 met the inclusion criteria. End-of-life care in ERs primarily involves symptom relief and optimal life-saving interventions. Barriers included the physical environment of the ER, healthcare professionals’ attitudes and communication challenges, and the complexities of patient-family dynamics. Facilitators included ER nurse support and environmental improvements that promoted patient comfort and privacy. This review highlights the need for improved understanding and delivery of end-of-life care in the ER settings, particularly for patients who die shortly after arrival. Future studies should validate assessment tools, develop educational programs for ER staff and families, and evaluate the impact of staffing levels on care quality. Limitations include variability in emergency care systems across countries and the lack of analysis on cultural or religious factors, which may affect the generalizability of the findings.

## Introduction and background

Globally, populations are experiencing demographic aging [1], and mortality rates are projected to continue rising, thereby potentially increasing emergency room (ER) deaths. In the United States, ER mortality rates rose slightly between 2018 and 2022 [2-3]. Similarly, in Japan, which has one of the largest aging populations, it was reported that of approximately 6.21 million individuals transported to hospitals in 2022, approximately 90,000 (1.5%) died shortly after arrival [4]. The ER, primarily an entry point for acute medical care and resuscitation, is increasingly facing end-of-life care demands. Advances in medical technology and pharmacotherapy have enabled people with multiple chronic conditions to remain in the community; however, they may require emergency transport when severely ill. Older adults with chronic illnesses often visit the ER in the final year of life [5]. Additionally, some patients, such as those with severe trauma or acute illnesses, cannot be saved despite medical intervention, making deaths in the ER inevitable. Therefore, integrating appropriate palliative and end-of-life care in ER settings is essential.

A “good death” is a key goal in end-of-life care and is highly individualized. It generally includes pain management, control of physical symptoms, collaborative decision-making, the presence of family members, and respect for the patient’s values and beliefs [6]. Preserving the patient’s dignity, enhancing satisfaction for both patient and family, and maintaining the patient's self-esteem are fundamental nursing responsibilities that support a good death [7]. In ERs, ensuring a good death for patients and their families remains a crucial aspect of end-of-life care.

However, providing end-of-life care in ERs poses unique challenges due to the diverse circumstances surrounding patients' deaths. Individuals may die suddenly from traffic accidents, drowning, cardiac arrest due to myocardial infarction, terminal cancer progression, or suicide. One of the main challenges is the lack of education and training programs on end-of-life care in clinical settings. Education and training for emergency department staff have been recognized as essential for providing appropriate end-of-life care to terminally ill patients with nonmalignant diseases, yet such opportunities remain insufficient [8]. Furthermore, the patient's wishes may be unknown or may conflict with those of their family. These factors can contribute to substantial stress for emergency nurses [9]. Moreover, families who experience bereavement in the ER often receive insufficient information, which can lead to confusion and feelings of guilt regarding the patient's death [10]. Given these challenges, it is imperative to clarify the characteristics of end-of-life care in ERs and systematically synthesize evidence to develop effective and appropriate interventions.

Previous research [11-12] has highlighted tailoring end-of-life care to patients’ and families’ specific circumstances to facilitate a good death. Key components include alleviating patient suffering from clinical symptoms, ensuring a peaceful passing, providing emotional support to families experiencing anxiety or grief, and communicating with individuals confronting unexpected loss. Moreover, managing a private, tranquil environment and providing postbereavement support are essential. A comparative analysis of end-of-life care in ERs in Middle Eastern and Western countries has shown that socioeconomic and cultural diversity and differences in religious beliefs shape care practices and patient experiences [13]. Major challenges include inadequate education and training programs for end-of-life care in clinical settings, along with variations in physicians' attitudes toward death and religious beliefs, which were identified as major issues. In Japan, sociocultural norms shape perceptions of a good death, and traditionally, it has been considered appropriate not to disclose the full truth to a dying individual [14].

Although the goal of end-of-life care is to facilitate a “good death,” its implementation and content vary by country and historical context. Moreover, the knowledge base lacks comprehensive systematization, and evidence is scarce on care provided to patients and their families when death occurs shortly after ER arrival. This study aimed to clarify the components of end-of-life in the ER, as well as the barriers and facilitators that influence its delivery.

## Review

Materials and methods

This scoping review was conducted in accordance with the methodological framework proposed by Arksey and O’Malley [15], further refined by Levac et al. [16], and expanded upon by the Joanna Briggs Institute [17]. We also adhered to the reporting guidelines outlined in the Preferred Reporting Items for Systematic Reviews and Meta-analyses extension for Scoping Reviews (PRISMA-ScR) guidelines for scoping reviews [18]. The protocol was registered in the University Hospital Medical Information Network Clinical Trials Registry (UMIN-CTR: UMIN000052075).

Review Questions

In this study, we formulated several key review questions: (1) What are the characteristics of patients and families of those who died in the ER? (2) What are the components and functions of end-of-life care as practiced and provided to these patients and their families, including symptom palliation? (3) Who are the providers responsible for delivering end-of-life care to patients who die in the ER and their families? (4) What indicators are used to evaluate the quality of end-of-life care in the ER? (5) What factors hinder or support the provision of end-of-life care in the ER?

Eligibility Criteria

The inclusion criteria were as follows: (1) study population (patients who died in the ER and their families), (2) concept (died in the ER without being admitted or transferred to the intensive care unit (ICU)), (3) context (end-of-life care provided in the ER), (4) study design (all studies reporting on aspects of end-of-life care for patients and families in the ER, including randomized controlled trials, cross-sectional, cohort, mixed methods, qualitative, quantitative longitudinal, and case studies published in academic journals), (5) language (no restriction), and (6) publication date (no restriction). We excluded reviews, case reports, opinion articles, letters, books, oral presentations, posters, and studies for which only the abstract was available.

Search Strategy

A comprehensive search was conducted across multiple databases, including PubMed, Cumulative Index to Nursing and Allied Health Literature (CINAHL), and Ichushi-Web of the Japan Medical Abstract Society. The search covered all available records from the inception of each database to November 22, 2024. In addition to database searches, relevant studies were identified through manual searches of key journals and by reviewing reference lists of selected articles. The search strategy was first developed in PubMed and then adapted for use in the other databases. No language restrictions were applied. The full search strings are presented in Table 1.

**Table 1:** Search terms CINAHL: Cumulative Index to Nursing and Allied Health Literature

Database	Search Terms
PubMed	Emergency Service, Hospital[mh] OR "emergency room*"[tiab] OR "emergency outpatient unit*"[tiab] OR "ER"[tiab] OR "accident and emergency"[tiab] OR "emergency service"[tiab] OR "emergency medical service*"[tiab] OR "emergency department"[tiab] OR "ED*"[tiab] OR "trauma center*"[tiab] AND Critical illness[mh] OR Heart Arrest[mh] OR Terminally ill[mh] OR Death[mh] OR Death, Sudden[mh] OR "critical illness*"[tiab] OR "critically ill"[tiab] OR "heart arrest"[tiab] OR "cardiopulmonary arrest"[tiab] OR "CPA"[tiab] OR "CPAOA"[tiab] OR "out-of-hospital cardiac arrest"[tiab] OR "death"[tiab] OR "die*"[tiab] OR "dying"[tiab] OR "terminally ill*"[tiab] OR "trauma death*"[tiab] OR "end of life"[tiab] OR "end-of-life"[tiab] AND Terminal care[mh] OR Palliative care[mh] OR "terminal care"[tiab] OR "palliative care"[tiab] OR "palliative treatment"[tiab] OR "end of life care"[tiab] OR "end-of-life care"[tiab] OR "EOLC"[tiab] OR "grief care"[tiab] OR "bereavement care"[tiab] NOT animals [mh] NOT humans [mh]
CINAHL	Emergency Service[mh] OR "emergency room*"[tiab] OR "emergency outpatient unit*"[tiab] OR "ER"[tiab] OR "accident and emergency"[tiab] OR "emergency service"[tiabl] OR "emergency medical service*"[tiab] OR "emergency department"[tiab] OR "ED*"[tiab] OR "trauma center*" AND Critical illness[mh] OR Critically Ill Patients[mh] OR Heart Arrest[mh] OR Terminally Ill Patients[mh] OR Death[mh] OR Death, Sudden[mh] OR "critical illness*"[tiab] OR "critically Ill Patient*"[tiab] OR "heart arrest"[tiab] OR "cardiopulmonary arrest"[tiab] OR "CPA"[tiab] OR "CPAOA"[tiab] OR "out-of-hospital cardiac arrest"[tiab] OR "death"[tiab] OR "die*"[tiab] OR "dying"[tiab] OR "terminally ill*"[tiab] OR "terminally ill patient*"[tiab] OR "trauma death*"[tiab] OR "end of life"[tiab] OR "end-of-life"[tiab] AND Terminal care[mh] OR Palliative Care[mh] OR "terminal care"[tiab] OR "palliative care"[tiab] OR "palliative treatment"[tiab] OR "end of life care"[tiab] OR "end-of-life care"[tiab] OR "EOLC"[tiab] OR grief care"[tiab] OR "bereavement care"[tiab] NOT Animals[mh] NOT humans[mh]
Ichushi-Web	Japanese search terms (used in actual query): 病院救急医療サービス/TH OR蘇生/TH OR初療室/TA OR救急外来/TA OR救命救急センター/TA OR高度救命救急センター/TA AND 死亡/TH OR突然死/TH OR致死的転機/TH OR心停止/TH OR突然死-心臓/TH OR院外心停止/TH OR末期患者/TH OR重症患者/TA OR終末期/TA OR エンドオブライフ/TA AND ターミナルケア/TH OR緩和ケア/TH OR グリーフケア/TH OR エンドオブライフケア/TA OR終末期ケア/TA OR悲嘆ケア/TA NOT 動物/TH NOT ヒト/TH
	English translation of search terms (for reference): Hospital emergency medical services/TH OR Resuscitation/TH OR emergency room/TA OR emergency outpatient department/TA OR emergency and critical care center/TA OR advanced emergency and critical care center/TA AND Death/TH OR Sudden death/TH OR Fatal outcome/TH OR Cardiac arrest/TH OR Sudden cardiac death/TH OR Out-of-hospital cardiac arrest/TH OR Terminally ill patients/TH OR critically ill patients/TA OR end of life/TA AND Terminal care/TH OR Palliative care/TH OR Grief care/TH OR end-of-life care/TA OR terminal phase care/TA OR bereavement care/TA NOT Animals/TH NOT Humans/TH

Selection and Data Extraction

The literature was uploaded to Rayyan (http://rayyan.qcri.org), where duplicates were removed. Of the eight reviewers (AF, YO, MH, MK, MM, MK, SY, and HS), two independently reviewed the titles and abstracts to identify potentially relevant studies. Publications that passed the initial screening were uploaded in full text to Rayyan, where two reviewers independently evaluated them based on the inclusion and exclusion criteria during the second screening phase. Any disagreements at any stage were discussed until a consensus was reached. If consensus could not be achieved, a third reviewer acted as a mediator. As is standard in scoping reviews, no formal bias assessment was conducted.

Data Collection and Charting

Data extraction was performed using a structured MS Excel (Microsoft Corporation, Redmond, Washington, United States) spreadsheet developed for this review. The data charting form included study characteristics (author name, year of publication, country, study design), participant characteristics (e.g., patient and family demographics), and key findings related to end-of-life care, including barriers, facilitators, and healthcare professionals involved. The form was pilot-tested on a subset of five studies to ensure consistency and clarity. Two reviewers (AF and HS) independently extracted the data, and discrepancies were resolved through discussion. If consensus could not be reached, a third reviewer (YO) was consulted. Data were managed using Microsoft Excel.

Data Synthesis

A narrative synthesis approach was employed to integrate the findings from the included studies. Extracted data were reviewed and interpreted collaboratively by multiple authors through iterative discussions. Data were coded based on their underlying meaning and grouped into conceptually similar categories to reflect key aspects of end-of-life care in the emergency room. Themes were not derived through formal thematic analysis; rather, meaning-based classification and consensus-building among authors guided the synthesis process.

Results

Selection and Inclusion of Studies

**Figure 1:**
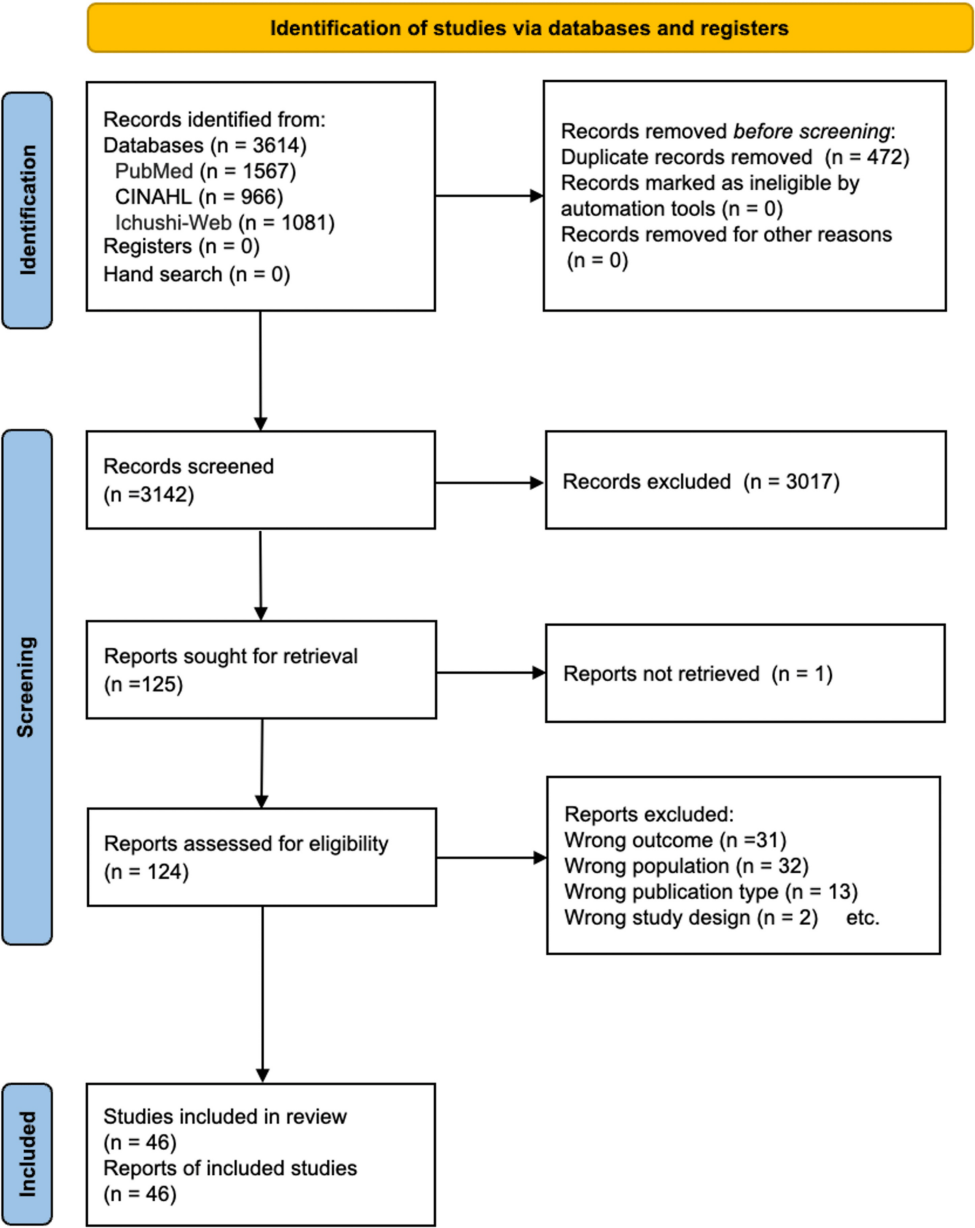
PRISMA 2020 flow diagram PRISMA: Preferred Reporting Items for Systematic Reviews and Meta-analyses; CINAHL: Cumulative Index to Nursing and Allied Health Literature

The search identified 3,614 articles. After screening titles and abstracts, 125 articles were shortlisted, though the full text of one was not accessible. Thus, 124 full-text publications were assessed for eligibility. Of these, 78 studies were excluded for various reasons, such as being unrelated to the concept or context. Ultimately, 46 studies met the inclusion criteria and were included in the review (Figure 1). 

Study Characteristics

The characteristics of the included studies are provided in Table 2. The oldest study was published in 1999, while 18 studies were published between 2020 and 2024. Thirteen studies were conducted in Europe, 10 in North America, 12 in Asia, eight in Oceania, two in the Middle East, and one study spanned three countries (Chile, the UK, and Spain). Regarding study design, 31 were observational, 11 were qualitative, three used a mixed-methods approach, and one was a case report. Only one intervention study was included-a before-and-after study evaluating the impact of multidisciplinary team interventions on the grief reactions of families of patients who died shortly after arriving at the ER.

**Table 2:** Characteristics of the included studies ED: emergency department; EOL: end of life

Authors, year	Country	Study design	Research question and objective
Ting et al., 1999 [19]	China	Before-and-after study	To study the early impact of bereavement and evaluate the effectiveness of bereavement care provided by a multidisciplinary team to close relatives of a sudden death, measured by the intensity of grief reactions
Tardy et al., 2002 [20]	France	Retrospective study	To determine the frequency, modalities of admission, and management of terminally ill patients who died on a stretcher in an ED
Harada, 2006 [21]	Japan	Grounded theory approach	To explore the grief experienced by a family following a sudden death in the ED over approximately 6 months
Heaston et al., 2006 [22]	USA	Questionnaire survey	To explore emergency nurses’ perceptions of the greatest obstacles and most supportive behaviors in providing care to dying patients in the ED
Beckstrand et al., 2008 [23]	USA	Questionnaire survey	To identify the obstacles and supportive behaviors in EOL care that have the greatest impact on patient care, as perceived by emergency nurses
Bailey et al., 2011 [24]	UK	Ethnographic methods	To understand the type of care provided to people nearing death, deceased individuals, and their bereaved families in emergency medical situations
Beynon et al., 2011 [25]	UK	Retrospective analysis	To determine the prevalence of palliative care needs experienced in the 3 months before death among individuals aged ≥65 years who died in the ED
Chan, 2011 [26]	USA	Interpretive phenomenological study	To describe the trajectories of how patients approached death in the ED to improve the care of patients in the EOL phase
van Tricht et al., 2012 [27]	France	Post-hoc subgroup analysis	To investigate the implementation of palliative care for patients who died in the ED
Beckstrand et al., 2012 [28]	USA	Mixed-method study	To determine emergency nurses’ suggestions for improving EOL care
Couilliot et al., 2012 [29]	France	Direct ethnographic-type observation at a distance and group interviews	To investigate the care practices surrounding EOL patients and the potential for providing palliative care in two ED short-stay units
Leak et al., 2012 [30]	USA	Descriptive analysis	To describe the characteristics of patients with cancer and their visits to the ED before death
Satake et al., 2015 [31]	Japan	Qualitative descriptive study	To identify the characteristics of nursing practices performed by skilled nurses for the families of terminal patients in tertiary emergency outpatient departments
Decker et al., 2015 [32]	Australia	Qualitative descriptive study	To examine how emergency nurses managed EOL care in the ED and responded to the needs of dying patients to facilitate a good death
Russ et al., 2015 [33]	Australia	Questionnaire survey	To investigate staff experiences and attitudes toward palliative care in a public metropolitan ED
Wolf et al., 2015 [34]	USA	Mixed-method design	To explore emergency nurses’ perceptions of challenges and facilitators in the care of patients at the EOL
Ka-Ming Ho 2016 [35]	Hong Kong	Cross-sectional descriptive survey	To evaluate the intensity, frequency, and magnitude of obstacles and supportive behaviors in providing EOL care as perceived by emergency nurses after implementing the EOL Care Pathway in Hong Kong
Nam et al., 2016 [36]	Korea	Descriptive correlational study	To determine the factors that influence healthcare providers’ attitudes toward EOL care in the ED within hospital settings
Granero-Molina et al., 2016 [37]	Chili, UK, Spain	Gadamer’s hermeneutic phenomenology	To explore and describe the experiences of physicians and nurses regarding the loss of a person’s dignity in EOL care in the ED
Hogan et al., 2016 [38]	Canada	An interpretive descriptive approach	To describe the experience of emergency nurses who provide care for adult patients who die in the ED to better understand the factors that facilitate care or challenge nurses as they care for these patients and their grieving families
Beckstrand et al., 2017 [39]	USA	Cross-sectional mailed survey	To identify suggestions that emergency nurses have for improving EOL care, specifically in rural EDs
Beckstrand et al., 2017 [40]	USA	Cross-sectional survey research design	To report the first-person experiences or stories of rural emergency nurses who have cared for dying patients and the obstacles they encountered while attempting to provide EOL care
Díaz-Cortés et al., 2018 [41]	Spain	Gadamer’s hermeneutic phenomenological approach	To explore and interpret physicians’ and nurses’ experiences regarding the conservation of dignity in EOL care for dying patients in the ED
Yates et al., 2018 [42]	UK	Retrospective cohort study	To examine patient characteristics, interventions provided, and outcomes in patients transferred to the hospital with ongoing cardiopulmonary resuscitation
Heymann et al., 2019 [43]	Switzerland	Monocentric retrospective analysis	To open our database of mortality figures to the scientific community to encourage further research on this often-overlooked aspect of acute care and to establish a foundation for future comparative studies
Economos et al., 2019 [44]	France	Retrospective cohort study	To assess the appropriateness of palliative treatments provided to patients who were actively dying at the time of care
Giles et al., 2019 [45]	Australia	Qualitative descriptive approach, Grounded theory	To explore nurses’ perceptions and experiences of caring for patients who die suddenly and unexpectedly in an ED setting
Cooper et al., 2020 [46]	USA	Prospective research study	To describe the implementation of ED Grief Support at our institution
Okawa et al., 2020 [47]	Japan	Analyze the free-text comments	To analyze free-text comments regarding grief care using text mining to clarify emerging trends
Vázquez-García et al., 2020 [48]	Spain	An observational, descriptive, cross-sectional study	To determine the clinical-epidemiological characteristics of patients who died in hospital EDs in the Autonomous Community of Aragon
Sadler et al., 2020 [49]	Saudi Arabia	A retrospective chart review	(1) To determine the incidence, nature, and illness trajectory of deaths occurring in the ED of a large tertiary hospital in Saudi Arabia; (2) to examine the extent to which EOL discussions had taken place before and during the final ED visit; (3) to analyze how aggressive the care was at the EOL; and (4) to determine the involvement of palliative care services
Martí-García et al., 2020 [50]	Spain	An observational, cross-sectional, descriptive, and analytical mixed-method study	To analyze the perceptions and experiences of relatives of patients dying from a terminal disease regarding the care provided
Ito et al., 2020 [51]	Japan	Qualitative content analysis	To explore the components of the quality of death for patients who die in the Japanese EDs and to provide a better understanding of EOL care geared toward achieving these patients’ experience of a good death
Kim et al., 2022 [52]	Korea	Single-center, retrospective cohort study	To clarify the characteristics of EOL care for actively dying patients in the ED at a tertiary hospital in Korea and the relevant factors influencing the provision of critical care and comfort care
Numanoi et al., 2022 [53]	Japan	Case studies	To examine the practicalities and challenges of family care in EOL situations for patients and families affected by COVID-19, focusing on bereavement care
Aquino et al., 2022 [54]	Australia	Cross-sectional survey research design	To identify the self-reported EOL care practices of emergency care nurses in Australia and the factors influencing EOL care provision
Ryan et al., 2022 [55]	Australia	Heideggerian phenomenological design	To gain insight into the structures of the phenomenon through the lens of emergency nurses when caring for older patients dying from traumatic injuries in an ED, with a focus on the concepts of lived time (temporality) and lived space (spatiality)
Kamimura et al., 2023 [56]	Japan	Questionnaire survey	To investigate whether the patient's family was able to recognize the consultation desk on the grief card and assess the ease of use of the help desk
Lee et al., 2023 [57]	Korea	Retrospective observational study	To investigate the epidemiologic characteristics of patients who died in the ED using a representative nationwide database and to identify the reasons for their ED visits
Omoya et al., 2023 [58]	Australia	Gadamer’s hermeneutic phenomenology	To determine Australian emergency doctors’ and nurses’ perceptions of their respective roles in providing EOL care
de Oliveira et al., 2023 [59]	Portugal	Cohort study	To identify the prevalence of palliative care needs in patients who die in the ED and to assess symptom control and the aggressiveness of care received by this patient population
Akman et al., 2024 [60]	Turkey	Cross‑sectional study	To investigate family members’ views on family presence during resuscitation and EOL care in the ED
Sweeny et al., 2024 [61]	Australia	Retrospective cohort study	(1) To describe the characteristics of people aged ≥ 65 years who died within 48 hours of ED presentation and (2) to describe how EOL practices were integrated into the care they received
Sirivarawuth et al., 2024 [62]	Thailand	Qualitative research	(1) To describe scenarios of the characteristics of critical EOL patients identified in the ED, as well as the perceptions and care needs of their family members; (2) to examine service delivery to improve quality of care and promote a good death from the perspective of emergency staff and bereaved families; and (3) to evaluate the model of care to enhance EOL experiences in the ED
Sweeny et al., 2024 [63]	Australia	Retrospective cohort study	To estimate the need for ED EOL care for people aged ≥ 65 years, describe the characteristics of those dying within 48 h of ED presentation, and compare patients in the ED with those who die elsewhere
McCallum et al., 2024 [64]	UK	Qualitative phenomenological study	To understand the experience of death and dying in a hospital ED

Characteristics of Patients Who Die in the ER, Their Families, and Care Providers

The characteristics of patients who died in the ER are provided in Table 3. The main causes of death among these patients included cardiovascular, respiratory, and neurological diseases. Other major causes were cardiac arrest, sepsis, accidents, and suicide. In addition, certain patients died in the ER due to natural causes or other illnesses. The age of these patients varied widely, ranging from 11 to 107 years. The length of stay in the ER ranged from 0.5 to 90.3 hours across the included studies. While some patients died shortly after arrival, others remained in the ER for extended periods. The relatives observing end-of-life care in the ER included spouses, children, and relatives. Healthcare professionals providing end-of-life care included nurses, physicians, chaplains, medical social workers, clinical psychologists, and paramedics.

**Table 3:** Characteristics of patients who die in the ER, their families, and care providers ER: emergency room

Domains	Extracted data
Cause of death	Cardiac arrests [24,35,30,57,61,63]
	Suicide [19,24]
	Accident (falls/traffic/assault) [19,24,48]
	Traumas [24,59,63]
	Severe burns [24]
	Sepsis [25,29,53,57,61,63]
	Shock [57]
	Neurological disease, including brain injury, neurologic bleeding, ischemic stroke, drug overdose, chronic neurological disease [24,29,48,49,52,57,59,61,63]
	Cardiovascular disease, including ischemic heart disease, aortic aneurysm and dissection, heart failure, chronic cardiovascular disease [24,25,29,38,48,49,52,53,57,59,63]
	Respiratory disease, including acute respiratory failure, respiratory infection, pulmonary embolism, chronic respiratory disease [25,29,30,38,48,49,52,57,61,63]
	Gastrointestinal and liver, including gastrointestinal bleed, vomiting, aspiration, asphyxiation, alcohol-related, liver failure, unable to eat, cachexia, dehydration [25,29,30,38,48,52,57,59,63]
	Renal failure, including other disorders of fluid, electrolyte, and acid-base balance [25,49,57,59,63]
	Natural diseases and others, including cancer, dementia, hypoglycemia/diabetes [19,20,29,30,38,48,52,57,61,63]
Age of the patients, year (min-max)	11-107 [20,21,25,29,30,43,44,46,48,49,52,56,57,59,61,63]
ER length of stay, h (min-max)	0.1-90.3 [20,25,44,57,59,61,63]
Characteristics of relatives: relationship to deceased person	Parent [19,46]
	Spouse/partner [19,46,51,62,64]
	Child [19,46,62,64]
	Sibling [19,46,51,62]
	Grandparents and others [46]
End-of-life care providers	Nurse [19,22,23,24,27,29,32,33,38,45,46,50,53,54,58]
	Physician [19,24,27,29,45,46,50,53,58]
	Chaplain [19,27,45,46]
	Medical social worker [19,27,45]
	Clinical psychologist [19,46]
	Technician [24]
	Paramedic [29]

End-of-Life Care Provided in the ER

The types of end-of-life care provided to patients who died shortly after arriving at the ER are provided in Table 4. End-of-life care in the ER primarily focused on symptom relief, including pain management, alleviation of respiratory distress, and treatment of delirium. Additionally, minimizing unnecessary procedures was recognized as an important aspect of end-of-life care. Ensuring that patients were not alone was a key priority, allowing them to spend time with their families. Conversely, in certain cases, continuing life-saving treatment and ongoing interventions were considered part of end-of-life care in the ER. Furthermore, in certain situations, patients were transferred from the ER to a quieter location to ensure privacy.

For families of patients who died in the ER, end-of-life care included the option to be present during resuscitation. Families were encouraged to spend time with the patient, and arrangements were made to facilitate visits. The presence of a healthcare professional to support grieving family members was considered an essential component of care. In addition, families were provided with information on available social support services following the patient’s death.

A few studies used assessment tools to measure the intensity of family grief reactions and to evaluate healthcare professionals’ attitudes toward end-of-life care. However, no standardized indicators are commonly used to evaluate the quality of end-of-life care provided to patients who die shortly after arrival at the ER and their families.

**Table 4:** End-of-life care provided in the ER ER: emergency room

Care recipient	Key components of care	Specific interventions/care practices
Patients	Relieve symptoms	Pain, nausea/vomiting, respiratory distress, delirium [26,33,44,51,61]
		Use of analgesic [26,27,29,44,61]
		Use of sedative [27,61]
		Reducing unnecessary procedures [26,28,41,44,51,62]
		Positioning change, oral care [27,29]
	Being with the patients	Ensure a place for patients and their families to spend time together [28,51,61,62]
		Increasing the time nurses have with patients [29]
		The nurse is just there [64]
		Intentionally communicate [45]
		Call the religious people [27,62]
	Providing care that meets the patient's wishes	Cultural considerations for end-of-life care [26]
		Checking the advance directive, discussion about end-of-life care [26,49]
	Be respectful	Maintain dignity, be respectful [27,42,51]
		Treat the remains with care [51]
	Doing our best for the patient	We will do our best to save lives [31,32,62]
		Continue with the treatment [40,55]
	Moving from ED	Move to a quiet place where you can have some privacy [32,41,45,61]
Families	Allocate time to spend with patients	Family presence during resuscitation [19,26,28,45]
		Family present to confirm death [19,29,62]
		Meeting management (such as time, environment, and appearance) [26,29,31,41,51,53,54,62]
		Lower the alarm volume, remove the monitor [29,54]
	Being with the families	Listen, care for family's feelings [31,47,62]
		Good communication with the families [45,61,62]
	Ensures the best care for patients	Tell the family that you did your best to treat the patient [26]
		Do not make the family feel guilty [51,62]
		Convey information properly [26,62]
		Ask about wishes for post-death care [33]
		Confirm the patient's wishes [51]
	Follow-up after death	Provision of information on post-death procedures [19,45,62]
		Providing information about social support [19,33,45,46,56,61]

Factors That Hinder and Support End-of-Life Care in ER

Three key factors were identified as barriers to providing end-of-life care in the ER: the ER environment, healthcare professionals’ perceptions, and the status of patients and their families. Specific barriers included a lack of privacy, limited time to fully engage with dying patients and their families, and the prioritization of care for other patients. Additionally, a lack of trust between healthcare providers and patients or families was recognized as an obstacle to effective end-of-life care.

Barriers identified by emergency medical personnel included a lack of knowledge and education regarding end-of-life care and the psychological burden associated with a patient’s death. Another major challenge was the lack of awareness of the patient’s imminent death by both the patient and their family (Table 5).

**Table 5:** Factors that hinder end-of-life care in ER ER: emergency room

Domains	Factors	Reported barriers
Environment of ER	Structure	No privacy [22,23,32-35,37,40,43,55]
		Too bright [34,64]
		Too hot [64]
		Family members cannot spend time with the patient at their leisure [34,36,38,47]
		This is not an environment for spending the end of a lifetime [24,32-34,38,64]
		Families cannot be present at the resuscitation scene [22]
	Priority and time constraints	Low priority [32,34,40]
		Busy, a lot of work [22,23,32-34,38,40,43,47,55,64]
		Other patient care takes priority [23,36,38,55]
		We must deal with the family [22,23,36]
		No time for a debriefing [43]
Healthcare professionals	Lack of knowledge and education	Lack of education [22,33,35,37,40]
		It does not respect the patient's wishes [43]
		No protocols [34,37]
		Pain management is difficult [40]
	Psychological burden	Difficulty changing our mindset [33,58]
		The burden of dealing with bereaved families [22-24,36,38,58]
	Relationship with the patient and family	No relationship with the patient and family [22,23,34,35,37,40,47,64]
	Continuation of futile treatment	Continuation of futile treatment [40,55]
Patients, families	Disagreement	Disagreements within families [36,37]
		Not realizing how close death was [22,37]
	Lack of social support	Lack of social support [40]

Two key facilitators of end-of-life care in the ER were support for ER nurses and an improved ER environment. Enhancing communication within the healthcare team and providing educational opportunities were considered essential for promoting better end-of-life care. In addition, ensuring privacy, maintaining a quiet environment, and establishing a support team or other systems to assist patients and families were identified as strategies to facilitate end-of-life care in the ER (Table 6).

**Table 6:** Factors that support end-of-life care in ER ER: emergency room

Domains	Factors	Reported support
Environment of ER	Structure	Privacy and a quiet space [22,28,33,38,41,64]
	Time constraints	Enough time for care [22,23,28,64]
		Limiting the length of stay for ER [41]
Healthcare professionals	System	Support team to help patients [28,38]
		Emergency physicians are qualified in palliative care [58]
	Communication within the team	Support from team members [38,54]
		Good communication within the team [23,47]
		Conference [47]
	Provision of educational opportunities	Education on end-of-life care [28,41]
		Recognize the value of end-of-life care [41,54]
	Care for patients and families	Pain management, positioning change, oral care [28,33,40]
		No needless procedures [41]
		Family presence during resuscitation [22,28,60]
		Good communication between the patients and the families [22,33,41,58]
		Helping the patient and family understand that the patient is close to death [22]
		Dignified care, communicating that you have done our best [22,23,28,33]

Discussion

In this scoping review, a qualitative synthesis was conducted based on 46 eligible studies that met the inclusion criteria and were available for data extraction. Causes of death in the emergency room include not only acute events, such as severe trauma, but also terminal cancer and chronic illnesses. The ER functions as a setting in which individuals from diverse backgrounds may spend their final moments. End-of-life care for patients who die shortly after arriving in the ER shares similarities with care provided in ICUs and general hospital wards. However, multiple barriers hinder its implementation, including a lack of privacy, the fast-paced environment, and the prioritization of critically ill patients. Additional challenges include limited knowledge and training in ER-specific end-of-life care, the absence of pre-existing patient-provider relationships, and the psychological burden on healthcare professionals managing patients and bereaved families. Facilitators of end-of-life care in the ER include structural adaptations to mitigate environmental constraints, effective communication within the medical team, and the provision of optimal treatment. However, standardized indicators for assessing the quality of end-of-life care and the quality of dying and death in the ER remain unclear. Consequently, there is a lack of research aimed at improving both the quality of end-of-life care and the overall patient management for those who die shortly after hospital arrival.

The content of end-of-life care provided in the ER and the providers of this care are similar to those in the ICU and general hospital wards [65]. However, ER healthcare professionals often lack the necessary knowledge and education to deliver adequate end-of-life care.

Systematic education on end-of-life care is available through programs such as the End-of-Life Nursing Education Consortium (ELNEC), which focuses on critical care settings [66]. Previous studies [67] have reported that structured education programs on end-of-life care improve nurses' knowledge and attitudes toward providing such care. ELNEC training has been reported to have a positive impact on emergency nurses as well; however, these programs are primarily designed for ICU patients and do not sufficiently address the needs of the families of patients who die shortly after arrival at the emergency department [68]. Therefore, the development of educational programs specifically tailored to end-of-life care in emergency settings is warranted.

Unlike the ICU, where end-of-life care is a recognized component of treatment, the primary goal in the ER is to provide immediate, life-saving interventions. In addition, structural limitations, such as the lack of private rooms, make it challenging to deliver end-of-life care in the ER. To resolve these challenges and improve care for dying patients and their families, it is necessary to develop systematic education programs tailored to the unique characteristics of the ER environment.

The causes of death among patients in the ER are diverse. Although cardiopulmonary arrest and accidents account for a considerable portion of ER deaths, many patients succumb to cancer and chronic diseases. As the number of ER visits continues to increase globally, the demand for end-of-life care in the ER is expected to rise [69]. In response, the integration of palliative care into emergency and intensive care settings has become essential. In the United States, the Improving Palliative Care in the ICU (IPAL-ICU) project aims to enhance the quality of palliative care in ICUs. Moreover, efforts to establish best practices for primary palliative care in ERs underscore the growing recognition of the importance of end-of-life care in emergency and critical care settings [70].

However, these initiatives largely focus on patients receiving prolonged treatment in the ICU and do not adequately address patients who are transported to the ER and die shortly thereafter. Consequently, best practices for this specific patient population remain underdeveloped, and effective care strategies are unclear. Future studies should aim to identify effective end-of-life care approaches for patients with cancer or chronic illnesses who die shortly after arrival, in addition to those who die from sudden events such as cardiopulmonary arrest or accidents. Moreover, given that the primary objective of ERs is to save lives, end-of-life care is often deprioritized in favor of emergency treatments for other critically ill patients. In addition to structural constraints, staff shortages are likely a key contributing factor. Previous studies have reported that changes in nurse staffing levels in the emergency department can increase nurses’ job satisfaction and improve the quality of care, as perceived by staff [71]. Based on these findings, it is possible that adjusting staffing levels in the ER could enhance the quality of end-of-life care; however, this has not been sufficiently verified. Future research should therefore examine the relationship between ER staffing and the quality of end-of-life care. Furthermore, improvements in staffing and resource management are essential to enhance the quality of death in ER settings.

To improve the quality of dying and death for patients in the ER, it is essential to evaluate the quality of end-of-life care. However, this study was unable to address this aspect, and appropriate indicators for assessing the quality of dying and death in the ER remain unclear. Various tools, such as the Quality of Dying and Death (QODD) scale, have been developed to assess the end-of-life experiences of patients in the ICU and their families [72]. The QODD, which has been translated into multiple languages and widely used in ICU settings, has yet to be validated for patients in the ER who die shortly after arrival. Consequently, intervention studies focused on improving the quality of dying and death in the ER are scarce. In the few existing before-and-after comparative studies, interventions have been assessed primarily based on family members' grief reactions without directly evaluating the quality of end-of-life care. Moreover, family satisfaction with ER end-of-life care remains unclear, as does the extent to which care provided aligns with the needs of patients and their families. Given these gaps, future intervention studies should focus on developing and implementing end-of-life care for patients who die shortly after ER admission.

Limitations

This review is the first to focus specifically on end-of-life care for patients who die shortly after arriving at the ER. Furthermore, by not imposing language restrictions, it enables comprehensive mapping of the available evidence.

However, this review has certain limitations. First, emergency medical systems vary across countries, thereby affecting transport criteria and legal frameworks for death confirmation. These variations may have influenced the study findings, as patient populations eligible for emergency transport differ by country. Consequently, the nature of end-of-life care provided may be affected by the characteristics of patients in the ER, requiring caution in interpreting the results. To address these gaps, further research examining end-of-life care in settings with diverse social, cultural, and religious backgrounds is warranted.

Second, this review does not analyze religious or cultural factors, primarily due to insufficient research data. Consequently, certain evidence-practice gaps may remain unaddressed, such as culturally specific post-mortem practices and gender-based considerations in patient-provider interactions.

Furthermore, this scoping review did not assess the quality of the evidence. Therefore, the results and implications derived from the included studies should be interpreted with caution.

## Conclusions

This study highlights the need for further research to assess the quality of end-of-life care in the ER, particularly for patients who die shortly after arrival. Although various tools exist for evaluating end-of-life care and the quality of dying, their applicability to patients in the ER who die soon after admission remains unverified. In addition, the lack of tailored education for ER healthcare professionals and insufficient staffing are major barriers to optimal end-of-life care. Future research should validate existing assessment tools for patients in the ER, develop educational programs to address the unique needs of patients in the ER and their families, and examine the impact of staffing levels on the quality of care. Addressing these gaps is essential to ensuring high-quality, patient-centered care for individuals who die in the ER.
